# Palliative stenting for malignant colorectal stenosis in the elderly

**DOI:** 10.1002/deo2.168

**Published:** 2022-09-30

**Authors:** Masashi Ohno, Atsushi Nishida, Kyohei Nishino, Hisashi Hirayama, Kenichiro Takahashi, Yukihiro Morita, Yuki Kishi, Yasuhiro Morita, Hiromichi Bamba, Hisanori Shiomi, Hirotsugu Imaeda

**Affiliations:** ^1^ Department of Gastroenterology and Hepatology Nagahama City Hospital Shiga Japan; ^2^ Department of Medicine Shiga University of Medical Science Shiga Japan; ^3^ Department of Gastroenterology and Hepatology Hikone Municipal Hospital Shiga Japan; ^4^ Department of Gastroenterology and Hepatology Nagahama Red Cross Hospital Shiga Japan; ^5^ Department of Surgery Nagahama Red Cross Hospital Shiga Japan

**Keywords:** colorectal cancer, elderly patients, palliative stents, prophylactic stents, self‐expandable metal stents

## Abstract

**Objectives:**

Self‐expandable metal stents are widely used for the treatment of malignant colorectal stenosis (MCS). In elderly individuals with MCS, self‐expandable metal stents are often used as a palliative treatment, but prophylactic stent placement is not recommended. We investigated the efficacy and safety of self‐expandable metal stents for the elderly in a palliative setting, specifically in a prophylactic setting.

**Methods:**

Elderly patients with MCS who received a palliative stent (the stent group) or palliative stoma (the stoma group) were retrospectively enrolled between April 2017 and June 2022, and the prognosis and complication rates were assessed. Additionally, patients in the stent group were divided into symptomatic and asymptomatic subgroups, and prognosis, stent patency, and complication rates were evaluated.

**Results:**

During the study period, 31 patients with a mean age of 85.4 years and 12 patients with a mean age of 82.0 years were enrolled in the stent and stoma groups, respectively. While overall survival and complication rates were comparable, the length of hospital stay was significantly shorter in the stent group. Of the 31 patients in the stent group, 16 asymptomatic patients received prophylactic stenting, which was not associated with increased complication rates.

**Conclusions:**

Palliative stents for MCS appear to be effective and safe even in the elderly, and thus, prophylactic stents can be considered for asymptomatic patients.

## INTRODUCTION

Malignant colorectal stenosis (MCS) is a serious condition mainly caused by colorectal cancer (CRC), which is the third most common cancer worldwide. It has been reported that 10%–18% of patients with CRC present with obstruction at initial diagnosis.^1^ If the stenosis is severe enough to cause colonic obstruction, an emergency surgery, such as colostomy or resection, has traditionally been considered.^2^ In recent decades, self‐expandable metal stents (SEMS) have been widely applied both for palliation of colonic decompression in inoperable patients and for preoperative decompression in surgical candidates.^3^, ^4^, ^5^, ^6^, ^7^, ^8^, ^9^, ^10^, ^11^ The European Society of Gastrointestinal Endoscopy recommends colonic stenting for palliation of malignant colonic obstruction.^12^ On the contrary, prophylactic stenting for asymptomatic MCS is not recommended,^12^ although MCS, regardless of the presence of obstructive symptoms, is associated with a higher risk of acute events that require emergency surgery.^13^

In developed countries where aging populations have increased, a higher number of patients with CRC are unable to tolerate colorectal resection because of poor physiological conditions, such as declining cardiopulmonary function and dementia. In these cases, regardless of colonic obstruction symptoms, palliative stenting including prophylactic stenting might be a promising treatment option. However, few reports have been published on the efficacy and safety of palliative stenting, including prophylactic stenting, for MCS in the elderly.

Our objectives were to compare the efficacy and safety of SEMS with decompressing stoma in a palliative setting and to compare the efficacy and safety of SEMS in the presence and absence of colonic obstruction symptoms.

## MATERIALS AND METHODS

### Study design

We conducted a retrospective study involving three hospitals located in the North and East Lake Area in Shiga Prefecture, Japan. All three hospitals have been appointed as a Regional Designated Cancer Care Hospital or a Regional Cancer Care Cooperation Hospital. The inclusion criteria were elderly patients (aged ≥65 years) who were diagnosed with MCS (with or without obstruction symptoms) and who had undergone palliative procedures between April 2017 and June 2022. All patients underwent both computed tomography and colonoscopy and had histologically proven adenocarcinoma. Patients who underwent primary resection before and after the palliative procedure were excluded. Patients with MCS due to extracolonic malignancy were also excluded.

The patients were first divided into two groups: the stent group and the stoma group. The stent group was further divided into two subgroups based on the presence of obstructive symptoms. The baseline information and clinical outcomes were collected from electronic medical records.

### Definitions

MCS was defined as the inability of a standard colonoscope (Olympus, Tokyo, Japan) to pass through the tumor. Determination of the presence of obstructive symptoms was based on the ColoRectal Obstruction Scoring System (CROSS).^10^ Specifically, a CROSS score of 4 was defined as asymptomatic MCS and less than 4 as symptomatic MCS. Prophylactic stenting was then defined as stenting in patients with asymptomatic MCS. Dementia was diagnosed according to the Diagnostic and Statistical Manual of Mental Disorders, Fifth Edition. Technical and clinical success was defined as the correct placement and expansion of the stent and the resolution of occlusive symptoms, respectively.^11^ Early complications were defined as those that occurred within 14 days after palliative procedures. Delayed complications were defined as those that occurred more than 14 days after palliative procedures.

### Palliative procedures

The selection of treatment was dictated by the physicians, taking into consideration the patient's prognosis, general condition, whether stoma care was possible, and whether close follow‐up was possible. All procedures in each group were performed by or were directly supervised by experienced endoscopists or surgeons. The SEMS used in this study were as follows: Niti‐S (Taewoong Medical, Seoul, Korea), HANAROSTENT (M.I. Tech, Seoul, Korea), and JENTLLY NEO (Japan Lifeline, Tokyo, Japan). In the stoma group, we selected patients who underwent palliative ileostomy or colostomy alone, but not primary resection, to match the background of patients in the stent group.

### Ethical considerations

This study was first approved by the ethics committee of Nagahama City Hospital (Nos. R3‐14 and R4‐2) and was subsequently approved by the ethics committee of each hospital.

### Statistical analysis

Categorical data are shown as numbers and percentages and were analyzed using Fisher's exact test. Numerical data are shown as means with standard deviation and were analyzed using an unpaired *t*‐test. The survival curves were determined using the Kaplan–Meier method and were analyzed with the log‐rank test. *p* < 0.05 was considered statistically significant. All statistical analyses were performed with GraphPad Prism (ver. 9.4.0; San Diego, CA, USA).

## RESULTS

We identified 31 and 12 patients who underwent palliative stenting and palliative stoma, respectively, for MCS from April 2017 to June 2022. The characteristics of patients in the stent and stoma groups are shown in Table 1. The mean age in both groups was over 80 years (85.4 ± 6.4 years in the stent group and 82.0 ± 8.6 years in the stoma group). No statistical differences were observed in age, gender, performance status, and tumor location; however, the stent group tended to have fewer cases of right‐sided colon cancer than the stoma group. All patients in the stoma group were diagnosed with stage IV unresectable CRC. Therefore, the clinical stage was significantly more advanced in the stoma group than in the stent group (*p* = 0.004). A significantly lower proportion of patients with dementia was observed in the stoma group compared with the stent group (*p* = 0.014). CROSS score was significantly lower in the stoma group (*p* = 0.003), indicating that obstructive symptoms were stronger in the stoma group. The improvement in CROSS score was also higher in the stoma group (*p* = 0.001).

**TABLE 1:** Patients’ characteristics

	**Stent (*n* = 31)**	**Stoma (*n* = 12)**	***p‐*value**
Mean age ± SD (years)	85.4 ± 6.4	82.0 ± 8.6	0.165
Gender			1.000
Female	18 (58.1%)	7 (58.3%)	
Male	13 (41.9%)	5 (41.7%)	
Performance status			0.460
0–2	8 (25.8%)	5 (41.7%)	
3–4	23 (74.2%)	7 (58.3%)	
Tumor location			0.092
Right	9 (29.0%)	7 (58.3%)	
Left	22 (71.0%)	5 (41.7%)	
Stage			0.004
II/III	14 (45.2%)	0	
IV	17 (54.8%)	12 (100%)	
Dementia (%)	16 (51.6%)	1 (8.3%)	0.014
CROSS score ± SD	2.6 ± 1.7	0.8 ± 1.5	0.003
ΔCROSS ± SD	1.3 ± 1.6	3.2 ± 1.5	0.001

SD, standard deviation; ΔCROSS was expressed as the change in CROSS score after palliative treatment.

**FIGURE 1:**
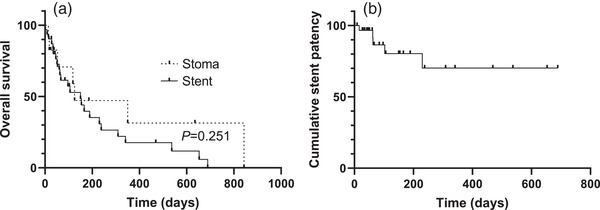
Kaplan–Meier curves of overall survival (a) and stent patency (b). The *p*‐value was calculated using the log‐rank test

Clinical outcomes of the stent group versus the stoma group during the mean follow‐up periods of 180.3 ± 33.9 days and 201.7 ± 78.7 days for the stent and stoma groups, respectively, are described in Table 2. All cases in both groups were technically and clinically successful. Early complications were not observed in either the stent or the stoma group. While no delayed complications occurred in the stoma group, 4 of 30 patients experienced delayed complications: one had perforation on day 230 and three had stent obstruction on days 16, 62, and 63. One patient in the stent group who experienced tumor perforation was treated conservatively and died 72 days later. Two of three patients with stent obstruction were successfully treated with the stent‐in‐stent technique; however, the other patient who experienced stent obstruction on day 16 died on day 18 of sepsis due to obstructive colitis. Notably, the length of stay in the stent group was significantly shorter than that in the stoma group (22.7 ± 20.9 days in the stent group and 42.3 ± 20.0 days in the stoma group, *p* = 0.008). No difference was observed in overall survival (OS) between the two groups (*p* = 0.251, log‐rank test; Figure 1a). The cumulative stent patency in the stent group is shown in Figure 1b. Two patients had stent patency of more than 600 days.

**TABLE 2:** Clinical outcomes of the stent group versus the stoma group

	Stent (*n* = 31)	Stoma (*n* = 12)	*p‐*value
Technical success	31 (100%)	12 (100%)	1.000
Clinical success	15/15 (100%)^a^	12 (100%)	1.000
Early complications	0	0	1.000
Delayed complications			
Total	4 (12.9%)	0	0.563
Stent dislocation	0	–	
Bleeding	0	0	
Perforation	1 (3.2%)	0	
Stent obstruction	3 (9.7%)	0	
LOS, days ± SD	22.7 ± 20.9	42.3 ± 20.0	0.008

Abbreviation:: LOS, length of stay; SD, standard deviation.

^a^ Only symptomatic patients were analyzed.

**FIGURE 2:**
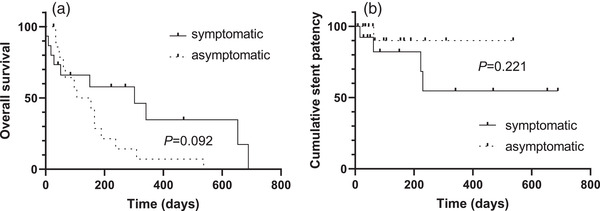
Kaplan–Meier curves of overall survival (a) and stent patency (b) in the symptomatic and asymptomatic subgroups within the stent group. *p*‐values were calculated using the log‐rank test

We next investigated the efficacy and the safety of prophylactic stenting in the stent group by comparing the clinical characteristics and outcomes of symptomatic and asymptomatic patients in that group. As shown in Table 3, no significant differences were observed in the characteristics between the two groups. No difference was observed in the delayed complication rate between the two groups, but the length of stay in the asymptomatic subgroup was significantly shorter than that in the symptomatic subgroup (Table 4). OS and the stent patency rate were not significantly different between the groups (*p* = 0.092 and *p* = 0.221, log‐rank test; Figure 2a,b).

**TABLE 3:** Patients’ characteristics of the symptomatic group and the asymptomatic group in the stent group

	**Symptomatic (*n* = 15)**	**Asymptomatic (*n* = 16)**	***p‐*value**
Mean age ± SD (years)	84.9 ± 6.7	85.8 ± 6.3	0.709
Gender, F/M			0.473
Female	10 (66.7%)	8 (50.0%)	
Male	5 (33.3%)	8 (50.0%)	
Performance status			1.000
0–2	4 (26.7%)	4 (25.0%)	
3–4	11 (73.3%)	12 (75.0%)	
Tumor location			0.113
Right	2 (13.3%)	7 (43.8%)	
Left	13 (86.7%)	9 (56.3%)	
Stage			0.480
II/III	8 (53.3%)	6 (37.5%)	
IV	7 (46.7%)	10 (62.5%)	
Dementia	6 (40.0%)	10 (62.5%)	0.289

Abbreviation: SD, standard deviation.

**TABLE 4:** Clinical outcomes of symptomatic group and asymptomatic group within the stent group

	**Symptomatic (*n* = 15)**	**Asymptomatic (*n* = 16)**	***p‐*value**
Technical success	15 (100%)	16 (100%)	1.000
Clinical success	15 (100%)	16 (100%)	1.000
Early complications	0	0	1.000
Delayed complications			0.3326
Total	3 (20.0%)	1 (6.3%)	
Stent dislocation	0	0	
Bleeding	0	0	
Perforation	1 (10.0%)	0	
Stent obstruction	2 (13.3%)	1 (6.3%)	
LOS, days ± SD	14.6 ± 10.8	30.4 ± 25.3	0.033

Abbreviations: LOS, length of stay; SD, standard deviation.

## DISCUSSION

CRC is predominantly a disease of the elderly and is a major cause of morbidity and mortality in elderly populations.^14^, ^15^ Elderly patients often have multiple comorbidities that are responsible for the increased risk of perioperative complications. It was reported that the incidence of postoperative morbidity and mortality increases progressively with advancing age.^14^, ^16^ Therefore, SEMS is widely used as palliative therapy for elderly patients with CRC. Previous studies showed that the use of palliative stents resulted in a shorter hospital stay compared with the use of palliative stoma.^2^, ^3^ However, no reports have clarified the efficacy and safety of prophylactic stenting in the elderly. In the current study, the patients who underwent palliative procedures for MCS were mostly very elderly patients with an average age of over 80. Because the selection of treatment is at the discretion of the physicians, there is a difference in patient background, particularly the presence of dementia, in the two groups. Although these biases require caution in interpretation, we found comparable prognosis and complication rates between the stent and stoma groups. Furthermore, stent placement was technically successful in all cases, including right‐sided CRC cases, in which placement is considered technically difficult.^17^ No postoperative complications were observed in the stoma group, which is presumably since the patients in the stoma group did not undergo primary resection but only stoma creation. On the contrary, 12.9% of patients in the stent group experienced complications, such as stent obstruction and perforation, which is similar to a previous report.^18^ Although two of three patients with stent obstruction could be treated with re‐stenting, the potential risk for serious complications should be considered prior to SEMS placement.

The percentage of patients with dementia was significantly lower in the stoma group than in the stent group. This is presumably because stoma creation was discouraged in patients with dementia due to their poor prognosis and difficulties in stoma care.^19^ In addition, significant differences were also observed in stage and the occurrence of colonic obstruction between the two groups. These differences in the patient background may have affected the resulting prognosis in both groups. However, since a better quality of life (QOL) is more important than longer survival in elderly patients,^20^ the efficacy of SEMS in reducing hospital stay might be a benefit for these patients.

Endoscopic obstruction, which is defined as colonic obstruction severe enough to prevent the passing of a colonoscope beyond the tumor, was reported to be associated with a higher risk of acute events that require emergency surgery.^13^ However, the European Society of Gastrointestinal Endoscopy does not recommend prophylactic stent placement because of the potential risk associated with colonic stenting.^12^ Considering that frequent colonoscopy in very elderly patients is associated with a high risk of complications, adverse events, and morbidity,^21^ prophylactic stentings are believed to be a treatment option in elderly patients if resection is not considered. Indeed, in this study, a comparable complication rate was seen between symptomatic and asymptomatic patients in the stent group, which indicates that prophylactic stenting is safe. We also observed the contrary results of OS and cumulative stent patency between the symptomatic and asymptomatic groups, although not statistically significant (Figure 2A,B). These results are probably due to the small sample size and bias in patient background.

To the best of our knowledge, this is the first study to compare the long‐term outcomes and prognosis of palliative stenting, especially prophylactic stenting, in the elderly. However, our study has several limitations. First, this was a retrospective study, which might have caused selection bias. Second, the sample size was small. Third, we did not accurately assess the degree of QOL and provide details of the comorbidities. Thus, additional multicenter prospective randomized controlled trials should be performed in the future.

In conclusion, our results show that palliative stenting is comparable with a palliative stoma in the elderly in terms of efficacy and safety. Moreover, prophylactic stenting is not associated with a higher complication rate. Therefore, SEMS may be a treatment candidate for elderly patients with CRC and stenosis even if they are asymptomatic.

## CONFLICT OF INTEREST

None.

## FUNDING INFORMATION

This work was supported by JSPS KAKENHI Grant Number JP21K15947.
